# Encouraging entrepreneurship in university labs: Research activities, research outputs, and early doctorate careers

**DOI:** 10.1371/journal.pone.0170444

**Published:** 2017-02-08

**Authors:** Michael Roach

**Affiliations:** Charles H. Dyson School of Applied Economics and Management, Cornell College of Business, Cornell University, Ithaca, NY, United States of America; Charles P. Darby Children's Research Institute, 173 Ashley Avenue, Charleston, SC 29425, UNITED STATES

## Abstract

This paper investigates how the encouragement of entrepreneurship within university research labs relates with research activities, research outputs, and early doctorate careers. Utilizing a panel survey of 6,840 science & engineering doctoral students at 39 R1 research universities, this study shows that entrepreneurship is widely encouraged across university research labs, ranging from 54% in biomedical engineering to 18% in particle physics, while only a small share of labs openly discourage entrepreneurship, from approximately 3% in engineering to approximately 12% in the life sciences. Within fields, there is no difference between labs that encourage entrepreneurship and those that do not with respect to basic research activity and the number of publications. At the same time, labs that encourage entrepreneurship are significantly more likely to report invention disclosures, particularly in engineering where such labs are 41% more likely to disclose inventions. With respect to career pathways, PhDs students in labs that encourage entrepreneurship do not differ from other PhDs in their interest in academic careers, but they are 87% more likely to be interested in careers in entrepreneurship and 44% more likely to work in a startup after graduation. These results persist even when accounting for individuals’ pre-PhD interest in entrepreneurship and the encouragement of other non-academic industry careers.

## Introduction

Entrepreneurial activity is increasingly encouraged on university campuses in the hope that it will foster the commercialization of scientific discoveries, stimulate job creation, and generate greater returns to federal investments in university research. To this end, the National Science Foundation and the National Institutes of Health have recently introduced programs such as the Innovation Corps (I-Corps) to foster entrepreneurial activity and prepare science and engineering graduate students for careers in entrepreneurship [1]. At the same time, there is considerable debate over whether science and entrepreneurship can coexist in research universities. Proponents contend that faculty and graduate students should embrace entrepreneurship as a means of broadening the impact of university research on society and economic growth, while also lamenting that academic norms discouraging entrepreneurship hinder such efforts. Opponents, on the other hand, express concerns that encouraging entrepreneurship may undermine the core mission of research universities by shifting attention away from fundamental research and toward commercial outcomes.

While progress has been made to reconcile these debates using patent and licensing data as proxies for academic entrepreneurship [2–4], such outcomes are influenced by disclosure requirements and universities’ efforts to commercialize research discoveries, and thus may not accurately capture the attitudes of university faculty and graduate students toward entrepreneurial activities [5, 6]. Moreover, patents have little bearing on the participation of academics as founders and employees of university startups. As a result, our understanding of the extent to which entrepreneurial activity is encouraged within university research labs and the possible consequences of such encouragement on academic research activity and career outcomes remain incomplete.

This paper investigates the encouragement of participating in entrepreneurship within university research labs across fields and universities, and how such encouragement relates with basic and applied research activities, publications and invention disclosures, and PhD student career interests and subsequent employment in academia and entrepreneurship. In addition to documenting considerable heterogeneity across fields in the encouragement of entrepreneurship, this study illustrates that encouraging entrepreneurship does not come at the expense of scientific research, providing new evidence that speak to concerns over whether encouraging entrepreneurship undermines academic research. Moreover, the results suggest that encouraging entrepreneurship influences certain PhD students to join startups as entrepreneurial employees without luring away PhD students interested in pursuing an academic career. These findings have important implications for federal and university policies to stimulate entrepreneurial activity and programs to prepare STEM PhD students for careers in entrepreneurship.

## Materials and methods

This study draws upon the Science and Engineering PhD Panel Survey (SEPPS), which was conducted by the author and a co-investigator in 2010, 2013 and 2016 [7]. The survey was approved by the Georgia Institute of Technology Institutional Review Board and validated by inviting a select sample of PhD students to complete the survey followed by an exit interview to probe students’ understanding of key questions and to solicit feedback on the instrument. Participation in the survey was voluntary and subjects consented by completing the survey.

The sample for the SEPPS includes 39 R1 U.S. research universities with doctoral programs in science and engineering that were identified using the National Science Foundation’s reports on earned doctorates [8]. The selection of universities was based primarily on PhD program size, while also ensuring variation in private/public status and geographic region (see S2 Table for a list of universities). The 39 universities in the sample produced roughly 40% of the graduating science and engineering doctorates in 2009 [8].

Approximately 30,000 individual names and email addresses were collected from university website to form the sample for this study. Emails were sent in February 2010 to invite PhD students to participate in the survey using the Qualtrics online software suite (www.qualtrics.com). When individual contact information was unavailable, emails were sent to administrators requesting that they forward a survey link to their graduate students. Overall, 84% of the responses were obtained through direct email contact and 16% were obtained through administrators. Adjusting for 6.3% undelivered emails, the direct survey approach achieved a response rate of 30% [9]. The final sample of 6,840 respondents is comprised of PhD students in the life sciences, chemistry, physics, and engineering, and excludes postdoctoral researchers.

Although the sample covers a range of institutions and fields, respondents are drawn from the population of approximately 100 “very high research activity” universities and may not generalize to students at other institutions. Nevertheless, as illustrated in Table 1, the respondents correspond to the distribution of PhD graduates reported in the NSF 2010 Survey of Earned Doctorates [10], suggesting that this sample is representative of U.S. PhD students in these fields.

**Table 1:** Comparison of NSF Survey of Earned Doctorates to SEPPS.

	NSF (2010)	SEPPS (2010)
Observations	27,137	6,926
Median age	30.5	27.2
Male	53.0%	60.8%
U.S. Citizen	65.7%	66.6%
Life sciences	44.0%	37.2%
Chemistry	8.9%	11.1%
Physics	7.3%	14.4%
Engineering	39.8%	37.3%

**Note**: NSF median age is at graduation, SEPPS median age is during PhD program.

### Measure of the encouragement of entrepreneurship

The measure of the encouragement of entrepreneurship is based on a question that asked PhD students “In your lab/department, to what extent are PhDs encouraged or discouraged to pursue the following careers?” using a 5-point Likert response scale that ranged from “strongly discouraged” (1) to “strongly encouraged” (5). Careers were rated independently and included university faculty with an emphasis on research (faculty), an established firm job with an emphasis on research or development (established firm R&D), and a startup job with an emphasis on research or development (entrepreneurship). The encouragement of a career in a startup is the featured measure in this study. Responses to this question are interpreted as reflecting PhD students’ perceptions of the acceptance within their lab of participating in entrepreneurial activities including founding or working in startups which are likely based on conversations with their faculty PI, other lab colleagues, and the employment outcomes of recent lab graduates. PhD students’ assessment of the encouragement of careers in entrepreneurship within their research labs is particularly relevant given the essential roles PhD students play as founders or key employees of university startups [11].

Although this is a direct measure of PhD students’ assessments of the encouragement of entrepreneurship in their labs, it is important to first probe its validity as a proxy for the encouragement of entrepreneurship more broadly. For example, one might be concerned that the encouragement of entrepreneurship simply reflects the more general encouragement of non-academic industry careers rather than the encouragement of entrepreneurship specifically. To examine the meaning of this measure, Table 2 reports ordered logistic regressions with the 5-point measure of the encouragement of careers in entrepreneurship as the dependent variable (Model 1), as well as the encouragement of careers in established firms (Model 2) and in academia (Model 3) for comparison. To the extent that these measures reflect the encouragement of distinct career paths, then they should exhibit different relationships with the independent variables that are correlates of the encouragement of different careers (see S1 Text for a detailed description of the survey questions used in this study). On the other hand, to the degree that the encouragement of careers in entrepreneurship and established firms correspond to the encouragement of non-academic industry careers more generally, then we should observe similar results across Models 1 and 2.

**Table 2:** Regression analyses of lab encouragement of different careers.

Methodology: Ordered logit	Lab encouragement of careers (5-point scale)		
Dependent variable	Entrepreneurship encouraged	Established firm encouraged	Academia encouraged
Model	(1)	(2)	(3)
Advisor has (co)founded company	0.42***	-0.16	-0.13
	[0.12]	[0.13]	[0.10]
Advisor is assistant prof.	-0.07	-0.06	0.17*
	[0.09]	[0.08]	[0.08]
Advisor is associate prof.	0.15	-0.11	0.09
	[0.08]	[0.07]	[0.08]
Entrepreneurship workshop/course	0.09***	-0.00	-0.00
	[0.03]	[0.02]	[0.02]
Level of basic research	0.08*	-0.03	0.16***
	[0.04]	[0.04]	[0.02]
Level of applied research	0.08**	0.08**	-0.06*
	[0.03]	[0.03]	[0.03]
Number of publications	0.01	-0.10*	0.14**
	[0.05]	[0.05]	[0.05]
Invention disclosure activity	-0.07	0.09	0.10
	[0.10]	[0.09]	[0.07]
Pre-PhD entrepreneurial interest	0.42***	-0.14**	-0.09*
	[0.05]	[0.05]	[0.04]
Pre-PhD academia interest	0.09	0.01	0.28***
	[0.07]	[0.08]	[0.04]
Lab encourages academia	-0.18***	0.65***	
	[0.05]	[0.04]	
Lab encourages est. firm	3.80***		0.84***
	[0.15]		[0.06]
Lab encourages entrepreneurship		3.78***	-0.21***
		[0.14]	[0.07]
Male	-0.01	0.01	-0.40***
	[0.05]	[0.06]	[0.06]
U.S. Citizen	-0.07	-0.08	0.30***
	[0.08]	[0.09]	[0.05]
Ph.D. start year	Incl.	Incl.	Incl.
38 field fixed effects	Incl.	Incl.	Incl.
39 university fixed effects	Incl.	Incl.	Incl.
Pseudo loglikelihood	-3735.77	-4006.80	-6478.13
Observations	6484	6484	6484

Robust standard errors clustered on PhD university in brackets.

*** p < 0.001.

** p < 0.01.

* p < 0.05.

Comparing coefficient estimates across models in Table 2 reveals significant differences between the three encouragement measures. For example, PhD students whose faculty advisor has been involved in founding of a company and PhD students who have participated in an entrepreneurship workshop or course are significantly more likely to report that entrepreneurship is encouraged in their lab. These variables are not significantly associated with the encouragement of careers in academia or established firms. In addition, PhD students who reported a pre-PhD interest in entrepreneurship also report that entrepreneurship is more strongly encouraged in their lab. While this relationship may reflect students with a predisposition toward entrepreneurship either sorting into more entrepreneurial labs or perceiving greater levels of encouragement, or both, it is nevertheless an important control for individual heterogeneity that may otherwise result in biased estimates if omitted. Although it is not possible to discern the causal direction of these relationships, the evidence suggests that PhD students’ assessments of the encouragement of entrepreneurship are systematically associated with correlates of entrepreneurial activity in meaningful and distinct ways from the encouragement of other careers.

## Results

This study first documents the encouragement of entrepreneurship across fields, universities, and faculty rank. It then investigates the relationship between encouraging entrepreneurship in university labs and PhD students’ basic research activities, as well as their number of publications and invention disclosure activities. Finally, this study examines the relationship between encouraging entrepreneurship and PhD students’ interests in academic and entrepreneurial careers during graduate training and their post-graduate employment outcomes in academia and in entrepreneurial firms.

### Encouragement of entrepreneurship across fields and universities

To simplify comparisons and data presentation, the 5-point scale for the encouragement of entrepreneurship was recoded into three categories that reflect whether entrepreneurship is “encouraged” (4 or 5 on the 5-point scale), “indifferent” (3, or neither encouraged not discouraged), or “discouraged” (1 or 2). In the aggregate, approximately 35% of PhD students report that participating in entrepreneurship is encouraged in their lab, 58% report that their lab is indifferent toward participating in entrepreneurship, and 7% report that participating in entrepreneurship is discouraged in their lab.

**Fig 1:**
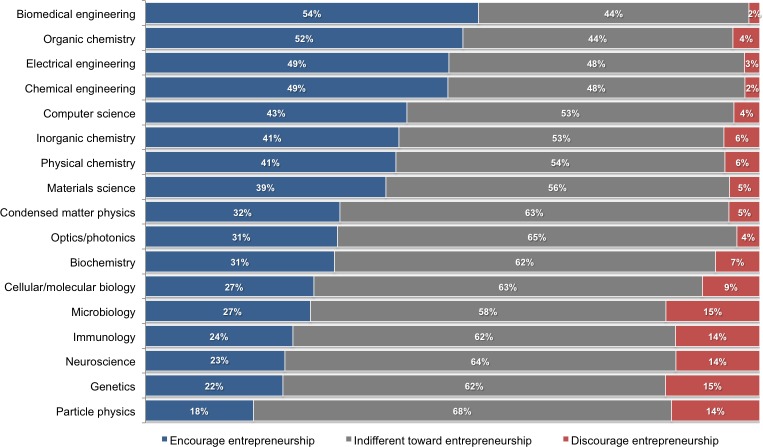
Encouragement of entrepreneurship across fields. Share of PhD students (n = 6,926) who report that participation in entrepreneurship is encouraged (blue), neither encouraged nor discouraged (indifferent in gray), or discouraged (red) within their lab.

Fig 1 illustrates the encouragement of entrepreneurship across select fields of science and engineering (see S1 Table for detailed results by field). The share of PhD students reporting that entrepreneurship is encouraged in their lab is greatest in engineering (47%) and chemistry (46%), and lowest in physics (26%) and the life sciences (24%). Only a small share of PhD students report that entrepreneurship is discouraged in their lab, ranging from 3% in engineering to 12% in the life sciences. Across many fields, a majority of PhD students report that their lab is indifferent toward (i.e., neither encourages nor discourages) participating in entrepreneurship, possibly indicating that entrepreneurship is not openly discussed in their lab. These patterns illustrate not only that there is considerable heterogeneity across fields in the encouragement of entrepreneurship, but also that entrepreneurship is more widely encouraged and less frequently discouraged than may be commonly expected.

**Fig 2:**
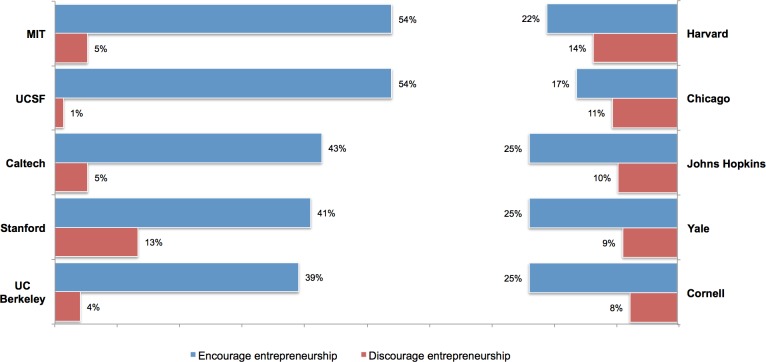
Encouragement of entrepreneurship across universities. Share of PhD students (n = 6,926) who report that entrepreneurship is encouraged (blue) or discouraged (red) within their lab by university. Universities with a reputation for entrepreneurial activity are on the left and peer universities are on the right.

Entrepreneurship is also encouraged to varying degrees across universities (S2 Table). As illustrated in Fig 2, the share of PhD students reporting that entrepreneurship is encouraged is greatest at universities with a history of entrepreneurial activity such as MIT (54%), Caltech (43%), Stanford (41%), and Berkeley (39%), while the share is notably smaller at peer research universities such as Chicago (17%), Harvard (22%), Yale (25%), and Cornell (25%). Although more “entrepreneurial” universities tend to have a greater share of faculty in engineering fields that are also more likely to encourage entrepreneurship per Fig 1, even within field there are meaningful differences between universities. For example, MIT and Harvard are roughly one mile apart yet differ notably with respect to the share of PhD students who report that entrepreneurship is encouraged in biochemistry (MIT 25%, Harvard 10%) and neuroscience (MIT 41%, Harvard 16%). While simply illustrative, these patterns suggest that more entrepreneurial universities differ from less entrepreneurial universities primarily in the degree to which entrepreneurship is encouraged rather than in the degree to which such activity is discouraged.

Ph.D. students were also asked about their faculty advisor’s entrepreneurial activities. Among PhD students in labs that encourage entrepreneurship, approximately 14% report that their advisor had (co)founded a company, compared to 8% in labs that are indifferent toward entrepreneurship and 5% in labs that discourage entrepreneurship. In addition, advisors with founder experience are more likely to be prominent senior faculty (16% endowed professors) rather than assistant (5%) or associate (7%) professors. When tabulated by the rank of the responding student’s faculty advisor there is no difference in the degree to which careers in entrepreneurship are either encouraged or discouraged. For example, 32% of Ph.D. students whose advisor is an assistant professor are in labs that encourage entrepreneurship, which is slightly lower than the share of students whose advisor is an associate (36%), full (33%), or endowed (38%) professor. At the same time, there is no difference by faculty advisor rank with respect to discouraging entrepreneurship, which ranged from 7–8%. These findings depart from the notion that junior faculty are more open to entrepreneurial activities relative to their senior faculty colleagues.

### Research activities and research outputs

A widely noted concern with encouraging entrepreneurship is that doing so may shift research activities away from fundamental scientific research and toward research with commercial outcomes. If so, then we would expect that PhD students in labs that encourage entrepreneurship would engage in research projects that are less basic and more applied in nature. To examine this relationship, this study draws upon a question that asked PhD students the degree to which their current research “contributes fundamental insights or theories (basic research)” and “creates knowledge to solve practical problems (applied research)”. Basic and applied research activities were assessed independently on a 5-point scale that ranged from “strongly agree” to “strongly disagree.” Each response was dichotomized to report the share of PhD students who agree (4 or 5 on the 5-point scale) that their current research activities include basic or applied research, respectively. Measuring basic and applied research separately allows for the observation of research projects that may consist of both fundamental and practical elements. Indeed, across fields 43% of PhD students report that their current research has both basic and applied elements, while 32% report that their research is primarily basic and 23% report that it is primarily applied.

Given the small share of PhD students who report that entrepreneurship is discouraged in theirs labs, the remaining analyses combine labs that discourage or are indifferent toward entrepreneurship into a single “not encouraged” category to more directly compare PhD students in labs that encourage entrepreneurship to students in labs that do not.

As reported in Table 3, the share of PhD students who report that their research is basic varies across fields (see S3 Table for detailed fields). However, within field the share is statistically indistinguishable between labs that encourage entrepreneurship and those that do not. For example, among life science PhD students in labs that encourage entrepreneurship, 93% report their current research as basic compared to 91% of students in labs that do not encourage entrepreneurship. In engineering, 57% of PhD students in labs that encourage entrepreneurship report their current research as basic compared to 55% of students in labs that do not encourage entrepreneurship. On the other hand, the share of PhD students whose research is applied is significantly greater in labs that encourage entrepreneurship across all fields. The difference is greatest in physics where 22% more PhD students report that their research has applied elements in labs that encourage entrepreneurship (57.8% compared to 47.3%), while the difference in engineering is only 6% (88.7% compared to 83.7%). In the life sciences, 14% more PhD students report that their research is applied in labs that encourage entrepreneurship (53.1% compared to 46.5%).

**Table 3:** Basic and applied research activities.

		*Basic Research*				*Applied Research*			
	Obs.	Entrepreneurship encouraged	Entrepreneurship not encouraged	*t-test*	*p-value*	Entrepreneurship encouraged	Entrepreneurship not encouraged	*t-test*	*p-value*
Life Sciences	2,543	92.7%	90.9%	-1.42	0.16	53.1%	46.5%	-2.88	0.01
Chemistry	756	75.4%	75.3%	-0.02	0.99	74.5%	67.6%	-2.09	0.04
Physics	990	83.4%	85.9%	1.03	0.31	57.8%	47.3%	-2.88	0.01
Engineering	2,551	57.0%	54.5%	-1.06	0.30	88.7%	83.7%	-3.05	0.01

These relationships are tested systematically through an ordered logistic regression analysis in Table 4. The dependent variable is the extent to which each PhD student’s current research makes basic (Model 1) or applied (Model 2) contributions on the 5-point scale, while the featured independent variable is the dichotomous measure of whether their lab encourages participation in entrepreneurship or not. All regressions are performed at the individual level of analysis and include controls for science or engineering field, university, PhD stage, and individual characteristics such as gender and nationality. Robust standard errors clustered on university are reported in brackets. The measure of whether a lab encourages careers in academia is also included for comparison and to control for unobservable lab characteristics that might be associated with basic research activity. For example, we would expect that labs that encourage academic careers perform more basic research relative to labs that do not encourage academic careers. Moreover, to examine whether the encouragement of entrepreneurship might simply reflect the more general encouragement of non-academic careers, Table 4 also reports result that control for encouraging careers in established firms. Since the majority of labs that encourage entrepreneurship also encourage established firms, this control reflects the additional variance explained by labs that encourage established firms but not entrepreneurship. Although these results should be interpreted as correlational and not causal, they nonetheless provide important insights into the relationships between the encouragement of entrepreneurship and other variables of interest such as basic and applied research activity, publications, and invention disclosures.

**Table 4:** Regression analyses of research activities and outputs.

Dependent variable	Basic research		Applied research		Number of publications			Invention disclosure		
Methodology	Ordered Logit		Ordered Logit		Poisson			Logit		
Model	(1a)	(1b)	(2a)	(2b)	(3a)	(3b)	(3c)	(4a)	(4b)	(4c)
Lab encourages entrepreneurship	-0.02	0.06	0.41***	0.31***	0.00	-0.01	0.06	0.27**	0.20*	0.18
	[0.06]	[0.09]	[0.07]	[0.07]	[0.04]	[0.04]	[0.06]	[0.10]	[0.10]	[0.11]
Lab encourages academia	0.39***	0.42***	-0.14*	-0.18**	0.06	0.05	0.07	0.01	0.03	0.02
	[0.04]	[0.05]	[0.07]	[0.07]	[0.04]	[0.04]	[0.04]	[0.09]	[0.09]	[0.10]
Lab encourages established firms		-0.13		0.15*			-0.09			0.03
		[0.09]		[0.07]			[0.06]			[0.10]
Basic research						0.05**	0.05**		0.01	0.01
						[0.01]	[0.01]		[0.04]	[0.04]
Applied research						0.04***	0.04***		0.37***	0.37***
						[0.01]	[0.01]		[0.05]	[0.05]
Male	0.09	0.09	0.14**	0.14**	0.14***	0.14***	0.14***	0.43***	0.41***	0.41***
	[0.05]	[0.05]	[0.05]	[0.05]	[0.03]	[0.03]	[0.03]	[0.08]	[0.08]	[0.08]
U.S. Citizen	-0.03	-0.03	-0.13**	-0.13**	-0.16***	-0.16***	-0.16***	-0.25**	-0.23*	-0.23*
	[0.06]	[0.06]	[0.05]	[0.05]	[0.04]	[0.04]	[0.04]	[0.10]	[0.10]	[0.10]
Ph.D. start year	Incl.	Incl.	Incl.	Incl.	Incl.	Incl.	Incl.	Incl.	Incl.	Incl.
38 field fixed effects	Incl.	Incl.	Incl.	Incl.	Incl.	Incl.	Incl.	Incl.	Incl.	Incl.
39 university fixed effects	Incl.	Incl.	Incl.	Incl.	Incl.	Incl.	Incl.	Incl.	Incl.	Incl.
Constant					1.01***	0.70***	0.69***	-2.66***	-4.02***	-4.00***
					[0.12]	[0.16]	[0.17]	[0.39]	[0.50]	[0.50]
Pseudo Loglikelihood	-7981.24	-7969.57	-8707.81	-8698.79	-12295.30	-12280.68	-12262.21	-2093.21	-2062.27	-2061.27
Observations	6752	6752	6752	6752	6713	6713	6713	6622	6622	6622

Robust standard errors clustered on PhD university in brackets

*** p < 0.001

** p < 0.01

* p < 0.05.

The results in Model 1a show that there is no significant relationship between labs that encourage entrepreneurship and the level of PhD students’ basic research activities, suggesting that within field encouraging entrepreneurship does not diminish basic research activity. Similarly, Model 1b shows that labs that encourage careers in established firms also do not conduct significantly less basic research. Model 2a shows a significant relationship between encouraging entrepreneurship and applied research activity. This relationship persists even when controlling for the encouragement of a career in an established firm in Model 2b which is only modestly related to applied research.

A related concern is that encouraging entrepreneurship may hinder or delay the publication of university research outputs, thereby resulting in fewer publications. Publications are measured using a survey question that asked each respondent to report their number of peer-reviewed publications. Table 5 illustrates that in most fields, PhD students in labs that encourage entrepreneurship have statistically the same number of publications as other students in labs that do not encourage entrepreneurship. Poisson regression estimates in Model 3a of Table 4 confirm the overall pattern that PhD students in labs that encourage entrepreneurship do not publish less. Model 3b includes measures of PhD students’ current levels of basic and applied research activities (inputs), which are important predictors of publications (outputs) with substantively identical results. Model 3c shows that labs that encourage careers in established firms also do not publish less. Thus, the evidence suggests that encouraging participation in entrepreneurship does not diminish scientific productivity.

**Table 5:** Publications and invention disclosure activity.

		*Number of Publications*				*Invention Disclosure*			
	Obs.	Entrepreneurship encouraged	Entrepreneurship not encouraged	*t-test*	*p-value*	Entrepreneurship encouraged	Entrepreneurship not encouraged	*t-test*	*p-value*
Life Sciences	2,543	1.85	1.81	0.41	0.68	7.4%	6.1%	-1.07	0.28
Chemistry	756	1.76	1.87	0.68	0.50	10.5%	9.1%	-0.62	0.53
Physics	990	1.63	2.04	2.39	0.02	8.7%	7.9%	-0.41	0.68
Engineering	2,551	1.52	1.45	-0.76	0.45	19.3%	13.6%	-3.29	0.01

One of the intended outcomes of encouraging entrepreneurship is the commercialization of university research discoveries. A first step in this process is the filing of an invention disclosure with the university’s office of technology transfer for research discoveries with commercial potential. Survey respondents were asked to provide information on the number of invention disclosures reported to their university technology transfer office. Across all fields, only 11% of PhD students reported that they had at least one invention disclosure, and among these students the mean number was 2.0. Given that the vast majority of PhD students did not have any invention disclosures, the measure was dichotomized at 1 or more invention disclosures to simplify the analysis. Table 5 reports the share of PhD students who had at least one invention disclosure, while Table 4 examines the likelihood that a PhD student has at least one invention disclosure.

Although the pattern within field is varied, Table 5 shows that overall a greater share of PhD students have invention disclosures in labs that encourage entrepreneurship compared to PhD students in labs that do not encourage entrepreneurship. However, the difference is significant only in engineering, where 42% more PhD students report that they have at least one invention disclosure in labs that encourage entrepreneurship relative to labs that do not (19.3% compared to 13.6%). Logistic regression analyses in Model 4a indicate that after controlling for field, university, and individual characteristics, PhD students in labs that encourage entrepreneurship are significantly more likely than other students to have at least one invention disclosure. Even after controlling for basic and applied research activity, which likely determine the commercial nature of a PhD student’s research, encouraging entrepreneurship is significantly associated with the likelihood of filing an invention disclosure. The results in Model 4c illustrate that PhD students in labs that encourage careers in established firms are not more likely to disclose inventions, indicating that encouraging entrepreneurship has a meaningful relationship with invention disclosures that is distinct from the more general encouragement of non-academic careers. However, within field regressions (see S5 Table) indicate that this relationship is largely in engineering, where PhD students in labs that encourage entrepreneurship are 41% more likely to have an invention disclosure. Although it is unclear whether encouraging entrepreneurship influences the likelihood of disclosing an invention or vice versa, the results provide evidence that encouraging entrepreneurship is associated with greater rates of invention disclosures, which are the foundation for university startups.

### Career interests and employment outcomes

In addition to the potential impact on university research activity and outputs, the encouragement of entrepreneurship may also influence PhD students’ career interests and employment outcomes. For example, concerns have been raised that encouraging entrepreneurship may lure PhD students away from careers in academia. On the other hand, encouraging entrepreneurship may legitimize entrepreneurship as a possible career path for science and engineering doctorates, leading to greater rates of new company formation and expanding the entrepreneurial workforce. For example, a key objective of the NSF I-Corps is to commercialize the outcomes of federally funded university research through teams that include the Principal Investigator and an Entrepreneurial Lead, typically a graduate student or postdoc who is part of the research team [12]. Encouraging entrepreneurship may lead to greater participation of recent PhD graduates as not only Entrepreneurial Leads, but also as early employees of university startups.

To consider the relationship between encouraging entrepreneurship and PhD students’ career interests, respondents were asked to report on the attractiveness of a set of possible future career paths, including academia and working in a startup, while putting aside job availability. By asking respondents to ignore labor market conditions, this measures is intended to capture PhD students’ underlying career interests without consideration for whether they can obtain a particular career or not. PhD students reported the attractiveness of academic and startup careers separately, providing a measure of their career interests independent of other possible careers. Respondents rated the attractiveness of each career separately on a 5-point scale from “extremely unattractive” (1) to “extremely attractive” (5). To simplify the analysis, the career attractiveness measures were dichotomized to distinguish between PhD students who find a particular career “attractive” or “extremely attractive” (4 or 5) from students who did not find that career attractive. The cutoff value of 4 is used because it reflects a substantively meaningful threshold in PhD students’ assessment of the attractiveness of each career.

As illustrated in Table 6, across science and engineering fields PhD students in labs that encourage entrepreneurship find academic careers just as attractive as students in lab that do not encourage entrepreneurship (see S4 Table for detailed fields). In addition, across fields a greater share of PhD students in labs that encourage entrepreneurship are attracted to careers in startups relative to students in labs that do not. The difference is greatest in the life sciences, where the share of PhD students attracted to working in a startup is 49% greater in labs that encourage entrepreneurship relative to labs that do not (60.9% compared to 40.6%), while in physics the share is 41% greater (65.4% compared to 45.9%) and in engineering it is 20% greater (72.3% compared to 60.9%). In chemistry, on the other hand, there is no distinguishable difference (53.8% compared to 53.5%).

**Table 6:** Academia and entrepreneurial career interests.

		*Academia career interest*				*Entrepreneurial career interest*			
	Obs.	Entrepreneurship encouraged	Entrepreneurship not encouraged	*t-test*	*p-value*	Entrepreneurship encouraged	Entrepreneurship not encouraged	*t-test*	*p-value*
Life Sciences	2,543	71.6%	69.3%	-1.17	0.24	60.9%	40.6%	-8.73	0.00
Chemistry	756	54.5%	50.8%	-1.00	0.32	53.8%	53.5%	-0.07	0.95
Physics	990	82.7%	75.8%	-2.16	0.03	65.4%	45.9%	-5.21	0.00
Engineering	2,551	70.2%	60.2%	-4.21	0.00	72.3%	60.9%	-5.00	0.00

Table 7 reports logistic regression estimates of the relationship between encouraging entrepreneurship and academic career interests. Model 1a shows that PhD students in labs that encourage entrepreneurship are more likely to report an academic career as attractive. Although this result may seem counterintuitive, encouraging academic careers has a greater relationship with the attractiveness of an academic career as one might expect, although the difference between the two coefficients is only modestly significant (χ^2^ = 2.85, p = 0.09). A particular econometric challenge with this analysis is that students with a predisposition toward entrepreneurship may both sort into labs that encourage entrepreneurship and have a greater attraction to a career in entrepreneurship, thereby biasing coefficient estimates. To account for PhD students’ predisposition toward entrepreneurship, Model 1b includes a binary variable that reflects students’ response to a question asking about their interest in entrepreneurship prior to starting the PhD program. The coefficient on this variable is negative and highly significant, indicating that PhD students with a predisposition toward entrepreneurship are less likely to find an academic career attractive. In addition, Model 1b includes PhD students’ levels of basic and applied research activity, respectively, which are likely associated with academic career interests. Indeed, both basic and applied research activities are associated with the attraction of an academic career, and this relationship is strongest for students whose research is also basic. Model 1c replaces the encouragement of entrepreneurship with the encouragement of careers in established firms, which is not significantly associated with an interest in an academic career. Taken together, the evidence suggests that encouraging entrepreneurship does not diminish PhD students’ interest in an academic career.

**Table 7:** Regression analyses of career interests.

Dependent variable	Academic career interest			Entrepreneurial career interest		
Methodology	Logit			Logit		
Model	(1a)	(1b)	(1c)	(2a)	(2b)	(2c)
Lab encourages entrepreneurship	0.16*	0.21**	0.24**	0.63***	0.42***	0.56***
	[0.07]	[0.07]	[0.09]	[0.07]	[0.08]	[0.10]
Lab encourages academia	0.33***	0.25***	0.26***	-0.38***	-0.25**	-0.20*
	[0.06]	[0.07]	[0.07]	[0.09]	[0.09]	[0.09]
Lab encourages established firms			-0.04			-0.21*
			[0.09]			[0.09]
Pre-PhD entrepreneurial interest		-0.62***	-0.62***		2.21***	2.21***
		[0.07]	[0.07]		[0.08]	[0.08]
Basic research		0.44***	0.44***		-0.00	-0.01
		[0.03]	[0.03]		[0.03]	[0.03]
Applied research		0.21***	0.21***		0.14***	0.14***
		[0.04]	[0.04]		[0.04]	[0.04]
Male	0.68***	0.76***	0.76***	0.57***	0.43***	0.43***
	[0.06]	[0.06]	[0.06]	[0.04]	[0.05]	[0.05]
U.S. Citizen	-0.63***	-0.77***	-0.76***	-0.61**	-0.25**	-0.25**
	[0.07]	[0.07]	[0.07]	[0.08]	[0.09]	[0.09]
Ph.D. start year	Incl.	Incl.	Incl.	Incl.	Incl.	Incl.
38 field fixed effects	Incl.	Incl.	Incl.	Incl.	Incl.	Incl.
39 university fixed effects	Incl.	Incl.	Incl.	Incl.	Incl.	Incl.
Constant	1.17***	-1.13***	-1.12***	0.23	-0.81**	-0.83**
	[0.22]	[0.31]	[0.31]	[0.25]	[0.33]	[0.33]
Pseudo loglikelihood	-3580.54	-3411.41	-3406.54	-3967.10	-3367.47	-3363.36
Observations	6286	6286	6286	6279	6279	6279

Robust standard errors clustered on PhD university in brackets

*** p < 0.001

** p < 0.01

* p < 0.05.

Model 2 reports logistic regression estimates for students’ interest in a startup career. Model 2a shows that after controlling for field, university, and individual characteristics, encouraging entrepreneurship is significantly associated with PhD students’ attractiveness of working in a startup. Indeed, the regression estimates indicate that PhD students in labs that encourage entrepreneurship are 87% more likely to find a career in entrepreneurship attractive, while students in labs that encourage academia are 69% less likely to be interested in working in a startup. These relationships persist even after controlling for students’ predisposition toward entrepreneurship (Model 2b), which is itself a strong predictor of students’ interest in working in a startup. The magnitude of this relationship also varies by field: in physics PhDs students in labs that encourage entrepreneurship are 114% more likely to be interested in working in a startup, in the life sciences students are 99% more likely, and in engineering they are 39% more likely. Model 2c illustrates that encouraging careers in established firms is negatively associated with startup career interests above and beyond the direct positive relationship associated with the encouragement of entrepreneurship (recall that the majority of labs that encourage entrepreneurship also encourage careers in established firms). Thus, encouraging entrepreneurship has a distinct and meaningful relationship with the attractiveness of working in a startup. Overall these results illustrate that PhD students in labs that encourage entrepreneurship do not find an academic career less attractive than their peers, while at the same time they are significantly more likely to find a career in a startup attractive.

Encouraging entrepreneurship may also influence PhD students’ subsequent post-graduate employment outcomes. To examine this, a follow-up survey was sent to respondents in 2013 and 2016 to inquire about their current employment, including whether they are employed in academia, have founded a company, or are employed in a startup or an established firm. The survey was supplemented through an exhaustive search of university websites and career profile websites (e.g. LinkedIn) that provided information on an individual’s employer and job title. External career profiles were identified by first searching for respondents by name and PhD university, such as “Jane Smith,” “PhD,” and “Duke University.” The match was verified by comparing field of study and years in the PhD program to the field and years in the PhD program reported in the survey. This approach yielded external data on employment outcomes for 77% of survey respondents. These data were used to supplement employment outcomes for non-respondents to the second wave of the survey. Combining both survey and external data provides employment outcomes for 92% of PhD student respondents.

**Fig 3:**
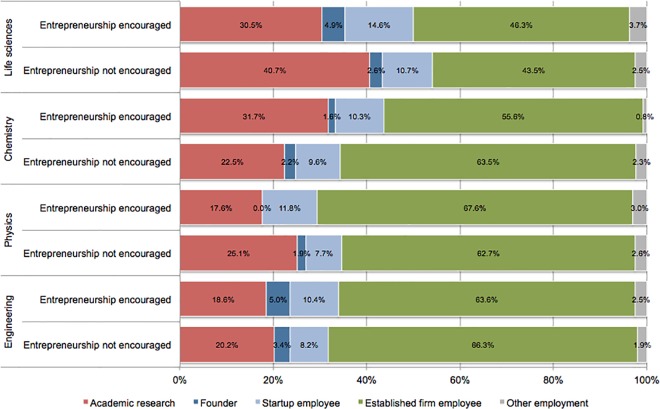
Post-graduate employment outcomes. Share of graduates (n = 2,608) in broad employment categories between labs that encouraged entrepreneurship or not during their PhD by field.

By 2016 nearly 2,800 PhD respondents had transitioned to fulltime employment in university research, a startup firm, an established firm, or as a founder of a new company. Academic employment was classified as individuals holding a tenure track university professor or non-tenure track university research position, excluding postdoctoral researchers. Startup employees were classified as individuals working in firms founded less than 10 years from the date of employment and with fewer than 500 employees, while other private sector employees were classified as individuals working in an established firm. Individuals who started a for-profit company after graduation, typically to commercialize a new technology, were classified as founders. Overall, 23% doctorates are employed in tenure-track or non-tenure track university research positions, 52% are employed in established firms, 7% are employed in startups, and 3% are founders. Fig 3 illustrates the share of individuals who are employed in each category after graduation.

Table 8 explores the effect of encouraging entrepreneurship during graduate school on subsequent employment outcomes while controlling for PhD students’ predisposition for entrepreneurship. Model 1a reports logistic regression results for the likelihood that a PhD student is employed in academia as either a tenure track or non-tenure track research faculty relative to other employment types (e.g., industry, national lab, etc.), while model 1b controls for individuals’ predisposition toward entrepreneurship as well as their predisposition toward academic careers. The results show that encouraging entrepreneurship has no influence on the likelihood of academic employment. Moreover, students with a predisposition toward entrepreneurship were significantly less likely to be employed in academia, while those with a predisposition toward an academic career were significantly more likely to be employed in academia. These results indicate that individuals’ predisposition toward different career paths rather than the encouragement of entrepreneurship influences academic employment.

**Table 8:** Regression analyses of post-graduate employment outcomes.

Dependent variable	Academic employee		Founder		Startup employee	
Methodology	Logit		Logit		Logit	
Model	(1a)	(1b)	(2a)	(2b)	(3a)	(3b)
Lab encourages entrepreneurship	-0.08	0.03	0.20	0.15	0.36*	0.58**
	[0.11]	[0.11]	[0.28]	[0.29]	[0.17]	[0.23]
Lab encourages industry				-0.06		-0.36
				[0.28]		[0.27]
Advisor has (co)founded company				0.39		0.04
				[0.26]		[0.12]
Pre-PhD entrepreneurial interest		-1.04***		0.48		0.28*
		[0.14]		[0.38]		[0.14]
Pre-PhD academic interest		0.47***		-0.13		0.06
		[0.09]		[0.21]		[0.14]
Male	-0.26*	-0.20	0.23	0.16	0.24	0.19
	[0.12]	[0.13]	[0.30]	[0.32]	[0.14]	[0.14]
U.S. Citizen	0.02	-0.07	0.19	0.24	0.94***	0.98***
	[0.18]	[0.19]	[0.31]	[0.35]	[0.16]	[0.17]
Ph.D. start year	Incl.	Incl.	Incl.	Incl.	Incl.	Incl.
38 field fixed effects	Incl.	Incl.	Incl.	Incl.	Incl.	Incl.
Constant	-0.79**	-0.75**	-3.58***	-3.89***	-3.71***	-3.79***
	[0.25]	[0.28]	[0.50]	[0.70]	[0.40]	[0.45]
Pseudo loglikelihood	-1372.52	-1306.58	-282.72	-279.80	-608.85	-605.01
Observations	2608	2608	1664	1664	1619	1619

Robust standard errors clustered on PhD university in brackets

*** p < 0.001

** p < 0.01

* p < 0.05.

Model 2 restricts the sample to respondents working in the private sector to examine the likelihood of becoming a founder relative to being an employee in a startup or an established firm. Neither the encouragement of entrepreneurship nor individuals’ predisposition toward entrepreneurship predict who become an entrepreneur. Model 2b also includes a binary variable indicating whether a PhD student’s faculty advisor has (co)founded a company to account for the possible influence of having an entrepreneurial role model, which has no effect on the likelihood of becoming a founder. These results indicate that encouraging entrepreneurship alone is insufficient in determining whether someone becomes an entrepreneur.

Model 3 further restricts the sample to only private sector employees (i.e., excludes founders) to investigate the likelihood that an individual is employed in a startup relative to an established firm. The encouragement of entrepreneurship significantly predicts startup employment, even when including individuals’ predisposition toward working in a startup, which also significantly predicts startup employment (Model 3b). PhD students in labs that encourage entrepreneurship are 79% more likely to work in a startup, while students who had a predisposition toward working in a startup are 32% more likely. PhD students whose advisor had (co)founded a company are not more likely to work in a startup. These results indicate that encouraging entrepreneurship may have a greater influence on subsequent employment in startups than individuals’ own predisposition toward entrepreneurship. In addition, it is important to note that non-U.S. citizens are significantly less likely to be employed in startups, despite the results in Model 2 of Table 7 that non-U.S. citizens are significantly more likely to be interested in working in a startup after graduation. Taken together, the results indicate that encouraging entrepreneurship does not diminish employment in academia, while it appears to influence science and engineering doctorates to join startups as employees rather than as founders [13, 14].

## Discussion

These findings illustrate that science and entrepreneurship coexist within research universities, with implications for federal and university policies to stimulate entrepreneurial activity and programs to prepare STEM PhD students for careers in entrepreneurship. First, although the body of evidence presented here is correlational and not causal, the results suggest that encouraging entrepreneurship does not come at the expense of universities’ fundamental research mission. More precisely, encouraging entrepreneurship does not diminish basic research or publishing, while it is significantly associated with invention disclosures, particularly in engineering. One implication is that when university research has both scientific and commercial outcomes, encouraging participation in entrepreneurship may indeed broaden the impact of university research on society through commercialization without diminishing its contribution to scientific advance.

Second, encouraging participation in entrepreneurship may have important implications for STEM doctorate career pathways and the entrepreneurial workforce. For example, given that far more PhD students prefer to join startups as employees rather than as founders [13], encouraging participation in entrepreneurship may do more to increase the pool of highly skilled scientific workers in the entrepreneurial labor force than to stimulate PhD students to start companies. As such, training programs should prepare PhD students for a wider range of entrepreneurial career paths and develop a broader set of skills beyond starting companies. In addition, encouraging entrepreneurship is likely to have a greater influence on PhD students with a predisposition toward entrepreneurship and commercialization, while it may have little influence on PhD students with a predisposition toward academic research [13]. As such, labs that encourage both fundamental research and participation in entrepreneurship will likely result in some PhD students pursuing opportunities to commercialize research discoveries while allowing other PhD students and faculty to direct their efforts toward research.

## Supporting information

S1 Table Encouragement of Entrepreneurship by Science & Engineering Field. (XLSX)

S2 Table Encouragement of Entrepreneurship by University. (XLSX)

S3 Table Encouragement of Entrepreneurship, Basic Research, and Invention Disclosures by S&E Field. (XLSX)

S4 Table Encouragement of Entrepreneurship and Career Interests by S&E Field. (XLSX)

S5 Table Regression Analyses by Life Sciences and Engineering Fields. (XLSX)

S1 Text Survey questionnaire. (DOCX)

## References

[1] NSF I-Corps: http://www.nsf.gpv/news/special_reports/i-corps; NIH I-Corps: http://sbir.cancer.gov/programseducation/icorps

[2] Azoulay P, Ding W, Stuart T. The Impact of Academic Patenting on the Rate, Quality, and Direction of (Public) Research Output. The Journal of Industrial Economics. 2009;LVII(4):637–76.

[3] Ding W, Choi E. Divergent Paths to Commercial Science: A Comparison of Scientists' Founding and Advising Activities. Research Policy. 2011;40(1):69–80.

[4] Bercovitz J, Feldman M. Academic entrepreneurs: Organizational change at the individual level. Organization Science. 2008;19:69–89.

[5] Agrawal A, Henderson R. Putting patents in context: Exploring knowledge transfer from MIT. Management Science. 2002;48(1):44–60.

[6] Owen-Smith J, Powell WW. To Patent or Not: Faculty Decisions and Institutional Success at Technology Transfer. Journal of Technology Transfer. 2001;26:99–114.

[7] The SEPPS was conceptualized and administered in equal collaboration with Henry Sauermann, who has coauthored other papers with the author using these data.

[8] National Science Foundation. Survey of Earned Doctorates. 2009.

[9] Sauermann H, Roach M. Increasing Web Survey Response Rates in Innovation Research: An Experimental Study of Static and Dynamic Contact Design Features. Research Policy. 2013;42(1):273–86.

[10] National Science Board. Science and Engineering Indicators 2010. Arlington,VA: National Science Foundation; 2010.

[11] Boh WF, De-Haan U, Strom RJ. Faculty and Students in Spin-offs: University Technology Transfer through Entrepreneurship. 2011.

[12] https://www.nsf.gov/news/special_reports/i-corps/teams.jsp; accessed December 6, 2016.

[13] Roach M, Sauermann H. Founder or Joiner? The Role of Preferences and Context in Shaping Different Entrepreneurial Interests. Management Science. 2015;61(9):2160–84.

[14] Roach M, Sauermann H. Founders and Joiners. Science. 2015;348(6240):1200–01. 10.1126/science.aab2804 26068826

