# Supplementary material for: Encouraging entrepreneurship in university labs: Research activities, research outputs, and early doctorate careers
Source: PLoS One. 2017 Feb 8;12(2):e0170444. doi: 10.1371/journal.pone.0170444 (PMC5298308; doi:10.1371/journal.pone.0170444)
Supplement: S1 Text — (DOCX) [file pone.0170444.s006.docx]

**S1 Text: Survey questionnaire**

**Encouragement of entrepreneurship**

**Q1: In your lab/department, to what extent are PhDs encouraged or discouraged to pursue the following careers?**

|  | Strongly disagree | Disagree | Neither agree nor disagree | Agree | Strongly agree |
| --- | --- | --- | --- | --- | --- |
| University faculty with an emphasis on teaching |  |  |  |  |  |
| University faculty with an emphasis on research or development |  |  |  |  |  |
| Government job with an emphasis on research or development |  |  |  |  |  |
| Startup firm job with an emphasis on research or development |  |  |  |  |  |
| Established firm job with an emphasis on research or development |  |  |  |  |  |

**Research activities**

**Q2: To what extent do you agree or disagree with the following phrases regarding the nature of your current research:**

|  | Strongly disagree | Disagree | Neither agree nor disagree | Agree | Strongly agree |
| --- | --- | --- | --- | --- | --- |
| My research contributes fundamental insights or theories (basic research) |  |  |  |  |  |
| My research creates knowledge to solve practical problems (applied research) |  |  |  |  |  |
| My research uses knowledge to develop materials, devices, or software (development) |  |  |  |  |  |

**Research outputs**

**Q3: How many of each of the following list you as an author or inventor? Please select 0 if none.**

|  | 0 | 1 | 2 | 3 | 4 | 5 | 6 | 7 | 8 or more |
| --- | --- | --- | --- | --- | --- | --- | --- | --- | --- |
| Conference proceedings/abstracts |  |  |  |  |  |  |  |  |  |
| Articles published or accepted in peer-reviewed journals |  |  |  |  |  |  |  |  |  |
| Research disclosures to university technology transfer office |  |  |  |  |  |  |  |  |  |
| Patent applications or issued patents |  |  |  |  |  |  |  |  |  |

**Career preferences**

**Q4: Putting job availability aside, how attractive do you personally find each of the following careers?**

|  | Extremely unattractive | Unattractive | Neither attractive nor unattractive | Attractive | Extremely attractive |
| --- | --- | --- | --- | --- | --- |
| University faculty with an emphasis on teaching |  |  |  |  |  |
| University faculty with an emphasis on research or development |  |  |  |  |  |
| Government job with an emphasis on research or development |  |  |  |  |  |
| Startup firm job with an emphasis on research or development |  |  |  |  |  |
| Established firm job with an emphasis on research or development |  |  |  |  |  |

**Pre-PhD career interests**

**Q5: Thinking back to when you began your PhD program in (year), how certain were you at that time that you wanted to pursue the following careers? Please provide a response for each.**

|  | Certain not to pursue | Unlikely to pursue | Uncertain whether to pursue or not | Likely to pursue | Certain to pursue |
| --- | --- | --- | --- | --- | --- |
| University faculty with an emphasis on teaching |  |  |  |  |  |
| University faculty with an emphasis on research or development |  |  |  |  |  |
| Government job with an emphasis on research or development |  |  |  |  |  |
| Startup firm job with an emphasis on research or development |  |  |  |  |  |
| Established firm job with an emphasis on research or development |  |  |  |  |  |

**Faculty advisor activities**

**Q6: To the best of your knowledge, has your advisor been involved in any of the following activities in the past three years?**

|  | Yes | No | Don’t know |
| --- | --- | --- | --- |
| Consulted for a company or other private enterprise |  |  |  |
| Patented an invention |  |  |  |
| Served as a member on scientific advisory board of a firm |  |  |  |
| Founded an entrepreneurial venture |  |  |  |
| Served as an executive (e.g., CEO/CSO/CTO) of an entrepreneurial venture |  |  |  |

**How many workshops, clinics, or courses on entrepreneurship (e.g., technology commercialization, licensing, founding a new company, etc.) have you taken while in your PhD program?**

*(ROW*: 0, 1, 2, 3, 4, 5, 6, 7 or more)

|  | 0 | 1 | 2 | 3 | 4 | 5 | 6 | 7 or more |
| --- | --- | --- | --- | --- | --- | --- | --- | --- |
| Conference proceedings/abstracts |  |  |  |  |  |  |  |  |
